# Lateral Boundary of Cirrus Cloud from CALIPSO Observations

**DOI:** 10.1038/s41598-017-14665-6

**Published:** 2017-10-27

**Authors:** Yunfei Fu, Yilun Chen, Rui Li, Fang Qin, Tao Xian, Lu Yu, Aoqi Zhang, Guosheng Liu, Xiangdong Zhang

**Affiliations:** 1 School of Earth and Space Sciences, University of Science and Technology of China (USTC), Hefei, 230026 P.R. China; 2 Department of Earth Ocean & Atmospheric Science, Florida State University, Tallahassee, Florida 32306 USA; 3 International Arctic Research Center and Department of Atmospheric Sciences, University of Alaska Fairbanks, 930 Koyukuk Dr, Fairbanks, Alaska 99775 USA

## Abstract

Due to the thinness and small scale of cirrus clouds, its lateral boundary may be missed by conventional passive remote-sensing techniques and climate models. Here, using satellite observations in June–August from 2006 to 2011, a global dataset for the cirrus cloud lateral boundary (CCLB) was established. The results indicate that the optical properties, such as the lidar backscatter, the depolarization ratio and the optical depth, sharply decrease from cloudy regions to clear-sky regions. There are significant regional differences in optical properties and height and thickness of the CCLB. Based on a quantitative estimation, the strongest longwave warming effects (>0.3 W m^−2^) are found near the Equator and over tropical continents. The global average longwave warming effect of the CCLB is at least 0.07 W m^−2^, which is much larger than some of the radiative forcings considered in the Intergovernmental Panel on Climate Change (IPCC) reports. Specifically, the CCLB in traditional “clear-sky” region may be totally missed by current models and IPCC reports, which contributes 28.25% (~0.02 W m^−2^) of the whole CCLB radiative effect, twice greater than contrail effect. It is recommended that the CCLB effect should be taken account in future climate models and the next IPCC reports.

## Introduction

Cirrus clouds regularly cover approximately 20% of the globe, providing significant radiative forcing (RF) on the Earth climate system^1–3^. It varies in altitude from a maximum cloud top of 17.6 km to a minimum cloud base of 6 km with an equivalent range in temperature from −82 to −7 °C, respectively^4^, and plays an important role in regulating the water vapor concentration in the upper troposphere and lower stratosphere^5^.

Cirrus clouds reflect shortwave solar radiation and absorb outgoing longwave radiation, and act as a thermostat, shielding Earth’s surface from solar radiation^6,7^. In most cases, the absorbed longwave radiation outweighs the reflected shortwave radiation^8^ and thus cirrus clouds have a net warming effect on the Earth. It is found that cirrus clouds with an optical depth greater than 0.06 have a significant longwave radiation absorption effect (~10 W m^−2^)^9^. Although there is large uncertainty in RF estimations of clouds, cirrus RF is generally calculated as a positive value; that is, cirrus clouds make both tropospheric and surface temperature increase^10–12^.

Satellite passive optical sensors, such as the Moderate Resolution Imaging Spectroradiometer (MODIS), can retrieve cirrus clouds during the daytime^13,14^, but the retrieval algorithm may misclassify up to 40% of thin cirrus clouds with an optical depth less than 0.3 as “clear sky”^15^. The CloudSat Cloud Profile Radar (CPR) can observe three-dimensional cloud structures, but still misses many of the thin cirrus clouds^16^. However, due to the high sensitivity and resolution of the Cloud-Aerosol Lidar with Orthogonal Polarization (CALIOP) onboard the Cloud-Aerosol Lidar and Infrared Pathfinder Satellite Observation (CALIPSO) satellite, there is a unique opportunity to comprehensively observe the thin cirrus clouds with range of optical depth from 0.01 to 5^16,17^.

The transition zone between cloud and clear sky is a region of dramatic change in terms of cloud optical properties, aerosol particles and water vapor. Several recent studies using ground-based radar and CALIPSO observations have focused on warm cloud and aerosols near the cloud edge, and found that reflectance, backscatter and optical thickness increase with decreasing distance from cloud^18–21^. The width of the transition zone still has large uncertainties due to different observation and calculation methods. Specifically, the width of the transition zone has been found to extend up to 15 km over ocean^21^ and up to about 6.4 km over land^22^, whereas other studies have found it to be less than 1 km for cumulus cloud^23^.

To the best of our knowledge, a few global observational studies have explored the relationship between the lateral boundary of cirrus clouds and their optical properties and the consequent RF. Using CALIOP measurements, Li *et al*. (2014) analyzed the lateral boundary layer of cirrus clouds in the east and south of China^22^ and estimated that the corresponding longwave RF is about 10 W m^−2^. Based on their conservative estimation, the RF induced by this cirrus cloud lateral boundary (CCLB) is at least 0.0047 W m^−2^ globally, which is not taken account in any global atmospheric circulations models, because there is no global model considers the lateral boundary of clouds. However, their RF result was calculated based on the assumption that coverage, width and RF of the CCLB are all fixed values. Furthermore, they assembled all the CCLBs into a “big round plate” to obtain the RF of this “plate”, that is, thousands of CCLBs were assemble into one enormous cloud, which may substantially underestimate the RF of the CCLB. Note that, because of the scan method (nadir-looking) of CALIOP, the transition zone is the wide regions around cirrus boundaries, as opposed to the exact 3D boundaries.

Accurate identification of CCLB can have great impacts on improving estimating global energy budgets and in turn global warming, helping climate model improvements that will reduce biases and uncertainties of climate simulation and projection. In this study, CALIOP data from 2006 to 2011 and an event-based method were used to construct a CCLB database and quantitatively address the following questions. (1) To what extent do the identification criteria affect the results? (2) What is the global distribution of the CCLB and its properties? (3) What contribution does the CCLB make to the Earth’s radiative energy budget?

## Methods

The standard CALIPSO-CALIOP Level 1B data and Level 2 vertical feature mask (VFM) data (version 3.01) for June–August from 2006 to 2011 were used to identify the CCLB. Level 1B data provide 532 nm attenuated backscatter profiles (*β*′_532_) and the depolarization ratio (*δ*_532_) at a horizontal resolution of 1 km and a vertical resolution of 60 m at altitudes of 8.3–20.2 km. VFM data provide cloud and aerosol classification at the same resolution as Level 1B data at altitudes of 8.3–20.2 km above mean sea level^17,24,25^. Only nighttime data were used in this study as there is lower noise and more reliable cloud detection due to weaker background illumination at nighttime^26^. Though many previous studies have simply used *β*′_532_, backscatter profiles (*β*_532_) were calculated from *β*′_532_ to reduce the error from two-way attenuation^22^.

It is difficult to quickly search for information on individual CCLB cases in original orbital data because of the large quantity of data. Therefore, it is necessary to eliminate useless data and establish a CCLB dataset. The event-based method first groups “cloud” and “clear sky” pixels, and then identifies the boundary points layer-by-layer to form an individual CCLB case. Specifically, the constraints for identifying the boundary points of each layer were improved from Li *et al*. (2014) and are listed below^22^.

1. “Cloud” and “clear sky” pixels should be wide enough (contiguous at least 45 km) with high reliability to ensure the location of the boundary from cloud to clear sky.
2. Cloud type should be “cirrus” with “confident” quality.
3. To avoid shifts in boundary locations caused by horizontal averaging, horizontal averaging of “cirrus” should be less than 5 km.
4. The cloud phase should be “ice”. Phase quality of more than 90% pixels should be “high confidence”.
5. *β*_532_ in the cloud should be significantly stronger than that in clear sky (minimum *β*_532_ in the cloud should be greater than the mean *β*_532_ in clear sky plus two standard deviations; clear sky here means the pixels identified as “clear sky” and 15 to 45 km away from the boundary point).

Based on the above criteria, the positions of the boundary points were obtained in each layer and then the proper cloud–clear sky interface cases were identified by the boundary points in continuous vertical layers.

**Figure 1:**
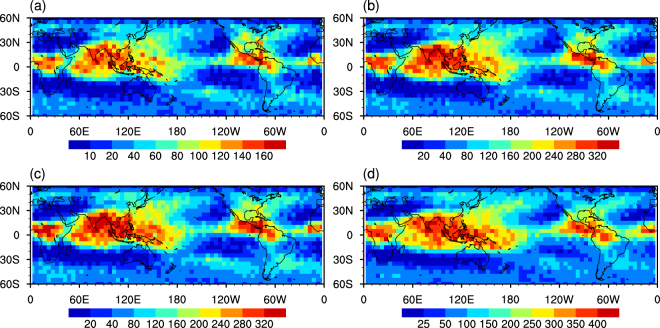
Distributions of the CCLB data points identified by different criteria groups in summer from 2006 to 2011: (**a**) criteria A; (**b**) criteria B; (**c**) criteria C; (**d**) criteria D.

Some atypical CCLB cases may be eliminated by our strict criteria. Possible regional differences in atypical CCLB cases will lead to an inaccurate global RF estimation. Therefore, it is necessary to examine the geographical distribution of the CCLB using different criteria. The above criteria, as the strictest criteria, are defined as criteria A. Criteria B excludes constraint (5); criteria C excludes constraint (5) and only requires phase quality better than or equal to “medium confidence” for constraint (4); criteria D, as the most relaxed criteria, only includes constraint (1) and (2) to guarantee the boundary is from “cirrus cloud” to “clear sky”. Figure 1 shows the geographical distribution of CCLB data points identified by the four criteria groups based on a 5° × 5° latitude × longitude grid. The sample sizes show a ratio of approximately 1:2:2:2.5 among the four groups with nearly the same distribution pattern. Overall, the distribution of CCLB cases is similar to the distribution of cirrus clouds, as revealed by Sassen *et al*.^27^. More CCLB cases are located in the tropical belt than the mid-latitudes as a result of anvils produced by deep convection in the intertropical convergence zone^28^. Typhoons over the western North Pacific enhance the troposphere-to-stratosphere water vapor transport^29^, and will also form injection cirrus. Few CCLB cases appear in northern Africa, southern Indian Ocean and eastern South Pacific, as well as regions with weak precipitation and convection over the Northern Hemisphere summer^30^. As a result, Criteria A was used in this study because of the strict requirements and the small effect on CCLB geographical distribution pattern. Although different criteria for identifying CCLB would lead to different CCLB properties and sample sizes, we believe that the current stage of using criteria A is the most appropriate, because lenient criteria may misidentify the CCLB and produce large uncertainty.

The SBDART (Santa Barbara DISORT Atmospheric Radiative Transfer) model was used to calculate the RF caused by the CCLB^31^. Cloud-top height, cloud-bottom height and optical depth of the CCLB were calculated on a 5° × 5° latitude × longitude grid, and then used as inputs to simulate the RF. As the differences between the CCLB and “clear sky” (RF, calculated by radiation fluxes of the CCLB minus clear-sky radiation fluxes) rather than the radiation fluxes themselves were our main interest, the uncertainties, such as atmospheric profile, should not significantly bias our conclusions^22^.

### Data availability

The datasets generated during and/or analysed during the current study are available from the corresponding author on reasonable request.

## Results

### Case study analysis

**Figure 2:**
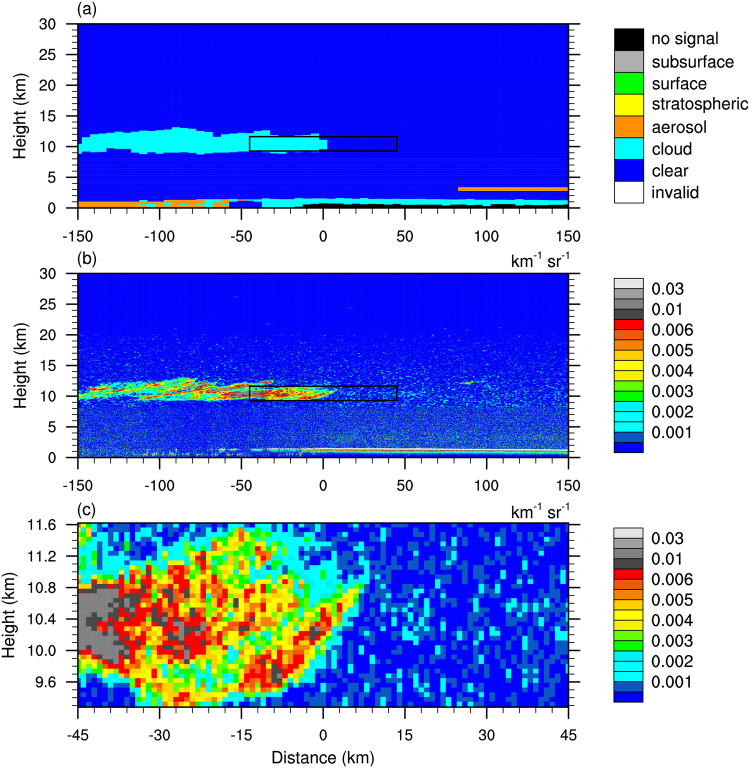
A cirrus cloud lateral boundary detected by CALIOP on 15 August 2007 over the East Pacific. (**a**) Classification of the type of targets based on the CALIOP vertical feature mask dataset; (**b**) β532; (**c**) β532 of the CCLB sample (black-box area in background field).

Figure 2 shows a prominent cloud–clear sky interface case identified using criteria A (see Methods) that occurred on 15 August 2007 over the East Pacific. It is apparent from Fig. 2a that a cloud layer occurred between 9 and 12 km altitude with a width of 150 km. On the right of the cloud layer, no cloud or aerosols were detected by CALIOP. It can be seen from Fig. 2b that the 532 nm backscatter (*β*_532_) in cloud is greater than that in clear sky, confirming the existence of the cloud. The cloud–clear sky interface identified by criteria A is shown in Fig. 2c.

**Figure 3:**
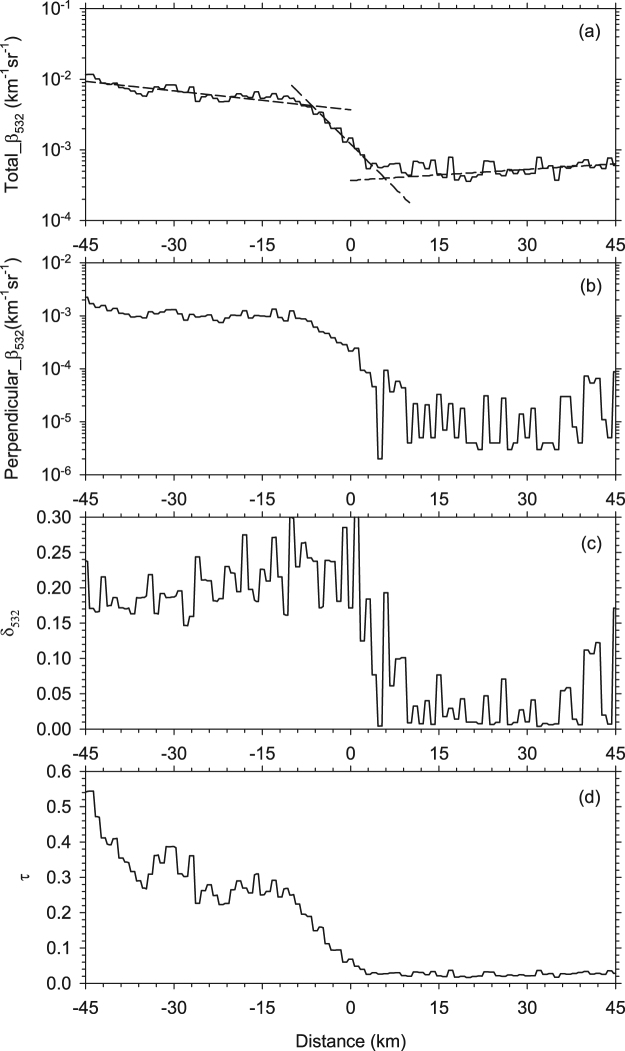
Optical properties of the cloud–clear sky interface sample shown in Fig. 1. (**a**) β_532_, (**b**) perpendicular β_532_, (**c**) δ_532_, and (**d**) τ. Distance zero indicates the boundary point.

**Figure 4:**
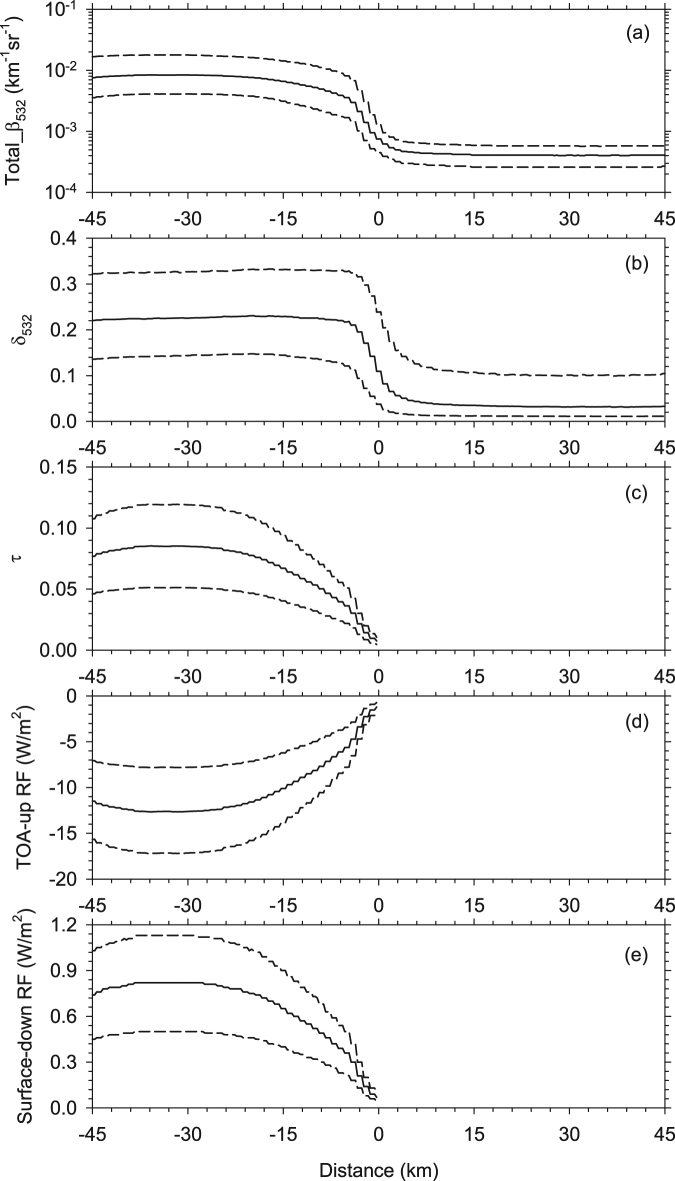
Optical properties and RF of the cloud–clear sky interface in summer from 2006 to 2011. (**a**) The median (solid line) and 75th and 25th percentile (dashed lines) β532; (**b**) the same as (**a**) but for δ532; (**c**) τ; (**d**) upward RF (cloud minus clear sky) at the top of the atmosphere; (**e**) downward RF (cloud minus clear sky) at Earth’s surface. Dashed lines in (**c**–**e**) mean uncertainty.

For the cloud–clear sky interface case in Fig. 2, *β*_532_ and perpendicular *β*_532_ were averaged layer-by-layer, and the depolarization ratio (*δ*_532_) was the ratio of perpendicular *β*_532_ to parallel *β*_532_, and the optical depth was calculated by integrating the volume extinction coefficient over the depth of the cloud layer. The results obtained from the preliminary calculation of optical properties are presented in Fig. 3. Interestingly, the distance to the boundary point is a dominant influence on *β*_532_. Figure 3a shows that there is a slight log-linear decrease from −45 to −6 km distance in the cloudy region, whereas *β*_532_ remains steady from 5 to 45 km distance in the clear-sky region. *β*_532_ drops sharply from 4 × 10^−3^ to 4 × 10^−4^ km^−1^ sr^−1^ in approximately 12 km between the cloudy and clear-sky regions. In addition, perpendicular *β*_532_ (Fig. 3b) shows a clear log-linear decreasing trend in the transition zone, and a similar trend to *β*_532_ in the cloudy region. However, perpendicular *β*_532_ in the clear-sky region is irregular, ranging from 10^−4^ to 10^−6^ km^−1^ sr^−1^, as it has a similar order of magnitude to noise. Therefore, *β*_532_ rather than perpendicular *β*_532_ was used to calculate the CCLB width. Before calculation, the cloud–clear sky interface case was roughly divided into three key regions according to distance to the boundary point: suspected cloud (−45 to −15 km), suspected transition zone (−5 to 0 km) and suspected clear sky (15 to 45 km). These typical distances were derived from the statistical analysis (Fig. 4a). On completion of this region segmentation, log(*β*_532_) for each distance in the three regions was calculated, followed by linear fitting of log(*β*_532_) and distance in the three regions. The intersections of the fitted line in the suspected transition zone with the fitted lines in the cloudy and clear-sky regions determined the CCLB width. The CCLB was then defined as the region between the two intersections, which is illustrated in Fig. 3a. *δ*_532_ shows a significant change at the CCLB (from ~0.2 to ~0.05). The optical depth (Fig. 3d) ranged from 0.25 to 0.5 with a mean of 0.35 in cloud, which may be partly missed by MODIS and CloudSat CPR^15,16^.

### Statistical analysis

Note that the linear fitting algorithm used to identify the boundaries of cloudy, clear, and transition zones in Fig. 3 was also used in the statistical analysis. To analyze the global statistical characteristics of the CCLB optical properties and RF, *β*_532_ and *δ*_532_ were averaged case-by-case, and the 75th, 50th and 25th percentiles of average *β*_532_ and *δ*_532_ were derived from almost 10^5^ effective cloud–clear sky interface cases. The percentile method can enhance the robustness compared with the average method^21^. As shown in Fig. 3a, a much smoother distribution is found from cloud to clear sky than for the cloud–clear sky interface case study above. A transition zone is found with a sharp decrease in *β*_532_, which reveals that the lateral boundary is not unique to the case study but is common in almost all cirrus clouds. The 25th to 75th percentile of *δ*_532_ ranges from 0.14 to 0.32 in the cloudy region as a result of multiple particle shapes in cirrus clouds, whereas nearly spherical particles appear more in clear sky with *δ*_532_ less than 0.05.

Median *β*_532_, cloud height and ambient temperature were used as inputs to derive the optical depth, the ice-water path and effective particle radius using the algorithms developed by Heymsfield *et al*.^32^. As shown in Fig. 4c, optical depth decreases from 0.08 (−20 km) to 0.02 (−3 km). In view of the small optical depth and scale of the CCLB, the associated RF may be missed by Clouds and the Earth’s Radiant Energy System (CERES)^33^. Based on the optical properties mentioned above, the RF from cloud to clear sky was retrieved. Note that only nighttime data were used to identify the CCLB in this study. Consequently, longwave radiation is the key RF used for calculations using the Santa Barbara DISORT Atmospheric Radiative Transfer (SBDART) model. The RF results are shown in Fig. 4d and e. The outgoing RF at the top of the atmosphere in the cloudy region is −15 to −10 W m^−2^, and the value sharply increases to 0 W m^−2^ through the transition zone. A possible explanation is that this negative outgoing RF (warming) is a result of absorption by cirrus clouds. According to some previous studies, cirrus clouds generally have a heating effect on the top of the atmosphere^34,35^. The trapped radiation then heats the cloud and atmosphere, and only a slight increase in longwave RF (Fig. 4e) is received on the Earth’s surface.

**Figure 5:**
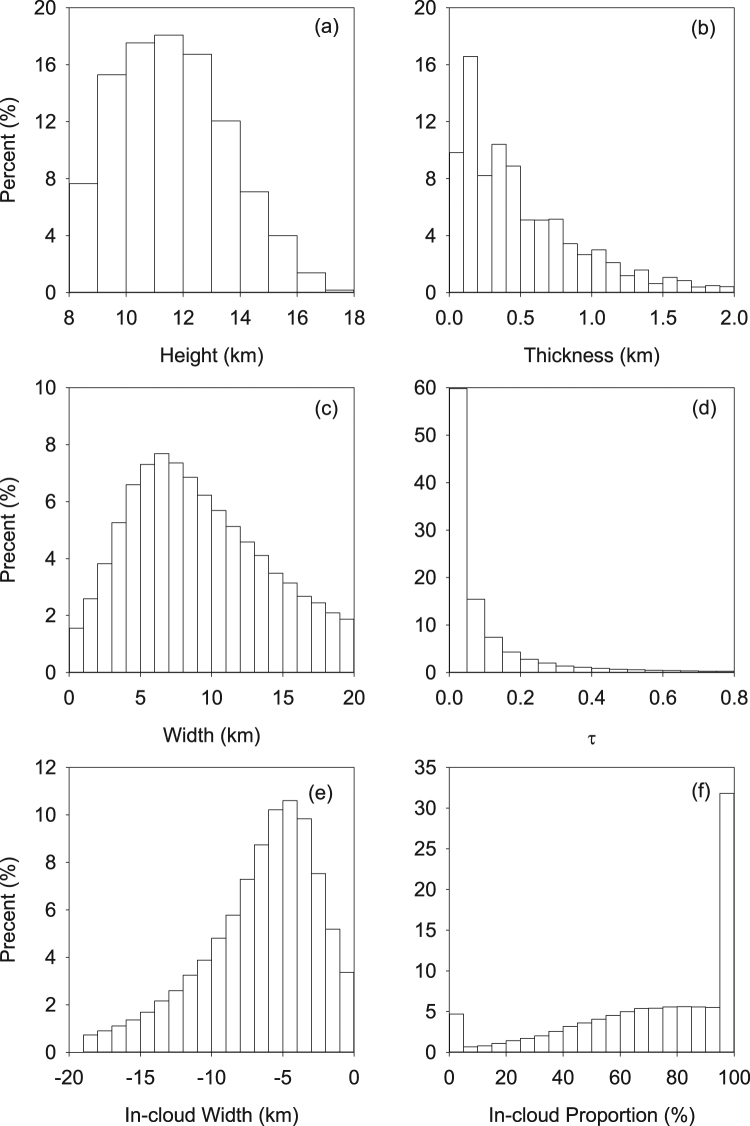
Probability distribution functions (PDFs) of the CCLB parameters in summer from 2006 to 2011. (**a**) Cloud height; (**b**) cloud thickness; (**c**) width; (**d**) τ; (**e**) location of the CCLB intersection in cloudy region; (**f**) proportion of the CCLB in cloudy region to the total CCLB.

The probability distribution functions (PDFs) for parameters of the CCLB are shown in Fig. 5. The cloud height was calculated by averaging cloud-top height and cloud-bottom height, and cloud thickness was the height difference between the cloud top and cloud bottom. It can be seen from Fig. 5 that more than 50% of cirrus clouds are between 10 and 13 km height. Less than 15% of cirrus clouds are above 14 km, and located at the upper troposphere. Cloud thickness shows an exponential distribution and thin cirrus clouds (cloud thickness less than 0.5 km) dominate by more than 50%. The proportion of CCLB width peaks at 5 to 8 km with more than 7% km^−1^, and then gradually declines to 2% km^−1^ at 20 km. It is possible that the wide variation in the CCLB width results from highly variable environmental conditions. Recently, Li *et al*. (2014) found a statistically significant correlation between the CCLB width and ambient temperature^22^. However, due to the spatial and temporal mismatch between reanalysis data and satellite observations, there is no general agreement on how these parameters affect the CCLB width. The optical depth in Fig. 5d was calculated by integrating the optical depth over the CCLB, and treating it as a constant to obtain the average optical depth for each CCLB case. For example, the optical depth in the case study (Fig. 2c) is about 0.15. As the CCLB is extremely thin and the optical depth is very small, such an assumption should not bias the findings in this study. More than half of the transition zones have an optical depth less than 0.05. A ground-based study by Kienast-Sjogren *et al*. (2016) at the Swiss high alpine site Jungfraujoch found that subvisible cirrus (*τ* < 0.03), thin cirrus (0.03 < *τ* < 0.3) and opaque cirrus (*τ* > 0.3) account for 43%, 46% and 11% of cirrus clouds, respectively^35^, which is similar to the magnitude of our findings. Since the light is almost fully extinguished within the thick clouds, their lidars could not specify the optical depth of the thickest cirrus clouds (τ > 3), which is almost the detection limit of CALIOP (τ ≈ 5). In addition, they concentrated on the whole cirrus clouds so that their τ is actually a little greater than our results.

As is shown in Fig. 3a, large part of CCLB occurs within cloudy region detected by CALIOP, and so it seems that this part has been considered in CALIOP-based studies. However, the properties of the CCLB are significantly different from the conventional “cloud” or “clear” pixels (see Figs 3a and Fig. 4). Therefore, it would be better to single out the CCLB as a separate part rather than only roughly divide pixels into “cloud” and “clear”. Then accurately calculating the location of the CCLB and identifying which part of CCLB is inside the conventional “cloud” region would be helpful for the future scene classification. Figure 5e shows the location of left intersection in cloudy region (about −6 km in Fig. 3a). On the cloud side of the cloud–clear sky interface, intersection of CCLB to actual “cloud” is located at about −5 km. ~30% CCLBs are totally located at conventional “cloud” and ~5% CCLBs are totally located at conventional “clear” (Fig. 5f).

**Figure 6:**
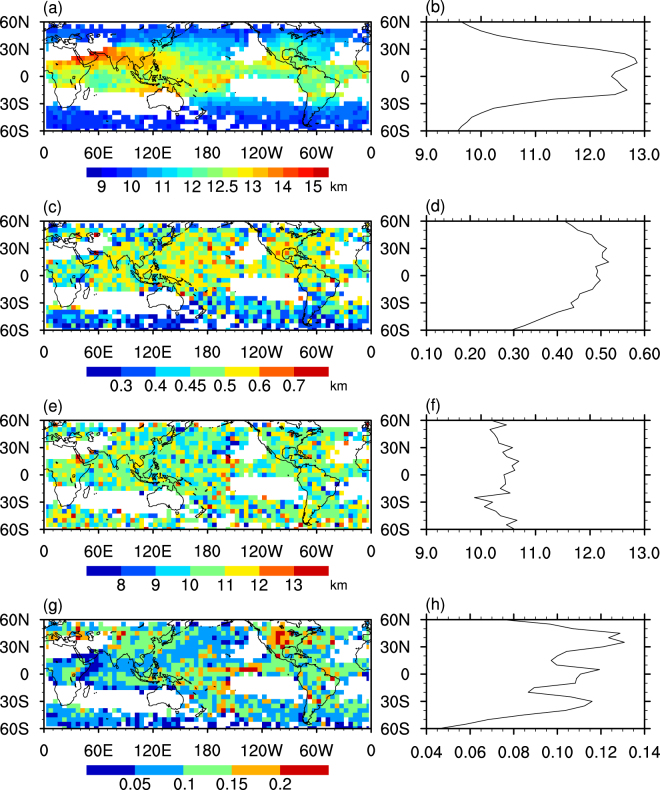
Distributions of CCLB parameters in the transition zone in summer from 2006 to 2011. Global distribution of (**a**) Cloud height; (**c**) cloud thickness; (**e**) width; (**g**) τ. latitudinal distribution of (**b**) Cloud height; (**d**) cloud thickness; (**f**) width; (**h**) τ. The white region means the number of CCLBs in each 5° × 5° grid is less than 20.

Li *et al*. (2014) found no significant differences in CCLB features among the three specific areas (eastern China, the East China Sea, and the South China Sea) of cirrus clouds in China^22^. However, regional variation would have been much more convincing if the global distributions had been explored. Figure 6 shows the average geographical distributions and latitudinal distribution of cloud height, cloud thickness, width and optical depth of the CCLB. Note that, to enhance the robustness of statistical results, only grids with at least 20 samples were used in the calculation. The highest CCLB height occurs in the tropics and subtropics, implying that a high CCLB appears to associate with a higher cloud top height. There are significant regional differences in cloud thickness, width and optical depth. For example, the CCLB cloud thickness is 0.5 to 0.6 km in the tropics and subtropics, but less than 0.45 km in the mid-latitudes in the Southern Hemisphere. This pattern is similar to that in Sassen *et al*.^27^, who found that cirrus cloud thickness is greatest in the tropics and decreases toward the poles^27^. Note that the thickness in our study is thinner than other reports, such as Sassen *et al*.^27^, because we only focus on the thickness at the lateral boundary rather than the whole cirrus cloud. Optical depths greater than 0.1 occur over East Asia, the Americas and central southern Pacific, which may be due to anvils produced directly by deep convection or southern storm tracks. These results indicate that regional variations in CCLB parameters do exist on a global scale, which leads to regional differences in RF.

**Figure 7:**
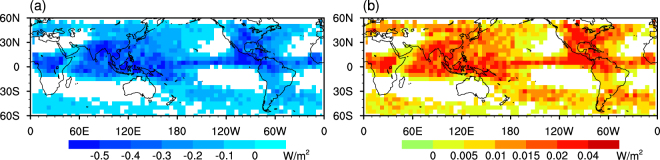
Distribution of the longwave RF induced by the CCLB in summer from 2006 to 2011. (**a**) Upward RF (cloud minus clear sky) at the top of the atmosphere; (**b**) downward RF (cloud minus clear sky) at the Earth’s surface.

Based on the CCLB properties calculated in Fig. 6, the outgoing RF at the top of the atmosphere and the surface downward RF were retrieved grid by grid. However, the calculation suffers from a lack of coverage of the CCLB. Li *et al*. (2014) assumed that the CCLB width is 6 km, and the global cirrus coverage is 25%^22^. This assumption reduces the computational complexity, but tends to overlook regional differences. To quantify the contribution of the CCLB in the regional radiative energy budget, the RF in each grid was computed using the following equation:

$${\rm{RF}}=\overline{R{F}_{c}}\times \,\frac{W\times Nu{m}_{c}}{Nu{m}_{v}\times R},$$

where $\overline{R{F}_{c}}$ is the mean RF when the CCLB exists, and $\frac{W\times Nu{m}_{c}}{Nu{m}_{v}\times R}$ is the CCLB coverage. Specifically, $W$ is the mean CCLB width, $Nu{m}_{c}$ is the number of CCLB cases (Fig. 1a), $Nu{m}_{v}$ is the total number of vertical feature mask (VFM) profiles and $R$ is the resolution ofthe VFM profile at altitudes of 8.3–20.2 km. Figure 7 shows the geographical distribution of upward RF at the top of the atmosphere and downward RF at Earth’s surface. It is almost certain that the existence of the CCLB reduces the outgoing radiation and increases the radiation absorbed by Earth’s surface^.^ The warming effect is greater than 0.3 W m^−2^ near the Equator and over tropical continents. According to the IPCC^36^^,^ RF is estimated as 0.01 (0.005 to 0.03) W m^−2^ for persistent (linear) contrails, with a medium confidence attached to this estimate. The RF of the CCLB is much larger than this recognized RF, but it is not considered fully in present climate models. We further calculated the global RF by averaging the whole grids in Fig. 7a, and found that the associated global RF is 0.07 W m^−2^. Furthermore, as CALIPSO cannot make observations at high latitudes^,^ the global RF could be larger than 0.07 W m^−2^. It is worth noting that the increase of $Nu{m}_{c}$ will lead to the increase of RF. Therefore, if the less strict criteria (such as Criteria B or C) was used to identify the CCLB, the larger global RF would be estimated than the above result (0.07 W m^−2^). As mentioned above, the less strict criteria may produce greater uncertainty for CCLB property estimation, even CCLB identification per se. We believe that with the development of instruments and algorithms, the estimated RF of CCLB will be more accurate than the current result.

As suggested by Lee *et al*.^15^, the longwave warming effect of tropical cirrus clouds is ~2.7 W m^−2^ at the top of the atmosphere, and ~0.6 W m^−2^ at the surface. Comparing with the RF of natural cirrus clouds, our results (greater than 0.3 W m^−2^ near the Equator, and 0.07 W m^−2^ for global at the top of the atmosphere) seem smaller an order of magnitude. However, it should be noted that conventional research may not – or not fully– consider the CCLB. Therefore, the RF caused by CCLB actually increases the uncertainty of cirrus RF, and needs to be accurately quantified.

Figure 5f shows the roughly one third of CCLBs lie entirely within cloud, and most CCLBs are halfway inside clouds. Is it possible that most CCLBs have been considered as cloud effects and less significant? We believe that some CCLB effects were indeed calculated in previous study, but please note that these CCLBs were treated as “clouds” rather than “transition zone” themselves. As was described, “cloud” and “transition zone” are different in optical properties so that misclassifying “transition zone” as “cloud” may introduce additional errors. To show the RF of CCLBs more accurately, a formula was designed to calculate the proportion of the inevitable missed CCLB, as well as the proportion of CCLBs in clear sky:

$${{\rm{P}}}_{m}=1-\,\frac{{\sum }^{}{P}_{I}}{Num}$$

Here, ${{\rm{P}}}_{m}$ is the missed proportion of CCLBs (the part in clear sky); ${{\rm{P}}}_{I}$ is the in-cloud proportion (x-axis in Fig. 5f, but for each CCLB); $Num$ is the amount of all CCLBs. After the investigation of the each CCLB, ${{\rm{P}}}_{m}$ is 28.25%, that is, at least $28.25 \% \times 0.07\approx 0.02$ W/m^2^ CCLB radiative effect has not been considered in any climate models and IPCC reports, which is still greater than contrail effect.

## Discussion

The purpose of this study was to identify the CCLB and then estimate its optical properties and associated global RF using nighttime CALIPSO observations in June–August from 2006 to 2011. A global CCLB dataset was established to increase the search speed for information on individual CCLB cases, and to analyze the CCLB parameters.

The CCLB width can be identified by linear fitting of log(*β*_532_) for the three key regions of cirrus clouds. It is hard for CERES to detect the small width (~10 km) of the CCLB, so the RF caused by the CCLB may be missed by current radiance observations. In addition, the optical depth of the CCLB is also so small that it may be partly missed by passive optical sensors such as MODIS or active microwave sensors such as CloudSat CPR. This study set out to gain a better understanding of the CCLB.

CCLB cases are mainly distributed in the intertropical convergence zone and tropical continents, which are high-frequency areas of high cloud^36^. The CCLB identification criteria affect the number of CCLB data points, but only slightly influence the geographical distribution pattern of the CCLB. The optical properties, such as 532 nm backscatter, the depolarization ratio and optical depth, all sharply decrease from the cloudy region to the clear-sky region in the CCLB. Globally, there are significant regional differences in the optical properties, and height and thickness of the CCLB.

The average longwave RF of the CCLB at the top of the atmosphere is −10 to −5 W m^−2^. In other words, the CCLB warms the Earth. This effect may have been missed by previous estimations of the radiative energy budget using passive remote sensing observations and climate models due to the thinness and small scale of the CCLB. Further research evaluated the regional differences in RF considering coverage of the CCLB, and found that the strongest warming effect occurred near the Equator and over tropical continents. Based on a quantitative estimation, the global RF of the CCLB is at least 0.07 W m^−2^, which is much larger than some forcings considered in the IPCC reports, such as persistent contrails from aviation (0.01 W m^−2^). Because the strict criteria were used in the current estimation, some cirrus-clear boundaries were not designed as CCLBs, which likely underestimated the global RF of the CCLB.

Specifically, CALIOP is nearly the most accurate sensor for cloud observation. We are confident that no model or IPCC reports consider the cloud transition zone in CALIOP-detect clear sky. Therefore, CCLBs in traditional “clear-sky” region may be totally missed, which contribute 28.25% (~0.02 W m^−2^) of the whole CCLB radiative effect, twice greater than contrail effect (~0.01 W m^−2^).

This study first established a global CCLB dataset, and then quantitatively investigated the global distribution of CCLB properties and the associated RF. Notwithstanding some uncertainties and limitations related to observations and modeling, for example, only nighttime data could hardly show the diurnal cycle and the relationship with deep convection, the main strength of this study is the quantitative estimation of the RF using regional statistics rather than assumed values. It would be interesting to assess the boundary radiative effects of other types of cloud. Also, it is recommended that the RF of the CCLB should be considered in future climate models, and “cloud boundary” should be identified as an independent subcategory in the future scene classification.

## References

[1] Liou KN (1986). Influence of cirrus clouds on weather and climate processes: a global perspective. Mon Weather Rev.

[2] Stephens GL, Tsay SC, Stackhouse PW, Flatau PJ (1990). the relevance of the microphysical and radiative properties of cirrus clouds to climate and climatic feedback. J Atmos Sci.

[3] Rossow WB, Schiffer RA (1999). Advances in understanding clouds from ISCCP. B Am Meteorol Soc.

[4] Platt C (1998). The optical properties of equatorial cirrus from observations in the ARM Pilot Radiation Observation Experiment. J Atmos Sci.

[5] Rosenfield JE, Considine DB, Schoeberl MR, Browell EV (1998). The impact of subvisible cirrus clouds near the tropical tropopause on stratospheric water vapor. Geophys Res Lett.

[6] Baker MB (1997). Cloud microphysics and climate. Science.

[7] Ramanathan V, Collins W (1991). Thermodynamic regulation of ocean warming by cirrus clouds deduced from observations of the 1987 El-nino. Nature.

[8] Fu Q, Liou KN (1993). Parameterization of the radiative properties of cirrus clouds. J Atmos Sci.

[9] Comstock, J. M., Ackerman, T. P. & Mace, G. G. Ground-based lidar and radar remote sensing of tropical cirrus clouds at Nauru Island: Cloud statistics and radiative impacts. *J. Geophys. Res*. **107** (2002).

[10] Rind D, Lonergan P, Shah K (2000). Modeled impact of cirrus cloud increases along aircraft flight paths. J. Geophys. Res..

[11] Meyer, R., Mannstein, H., Meerkotter, R., Schumann, U. & Wendling, P. Regional radiative forcing by line-shaped contrails derived from satellite data. *J. Geophys. Res*. **107** (2002).

[12] Minnis P, Schumann U, Doelling DR, Gierens KM, Fahey DW (1999). Global distribution of contrail radiative forcing. Geophys Res Lett.

[13] Gao BC, Yang P, Han W, Li RR, Wiscombe WJ (2002). An algorithm using visible and 1.38-mu m channels to retrieve cirrus cloud reflectances from aircraft and satellite data. IEEE T Geosci Remote.

[14] Gao BC, Yang P, Li RR (2003). Detection of high clouds in polar regions during the daytime using the MODIS 1.375-mu m channel. IEEE T Geosci Remote.

[15] Lee J, Yang P, Dessler AE, Gao BC, Platnick S (2009). Distribution and radiative forcing of tropical thin cirrus clouds. J Atmos Sci.

[16] Stein T, Delanoe J, Hogan RJ (2011). A comparison among four different retrieval methods for ice-cloud properties using data from CloudSat, CALIPSO, and MODIS. J Appl Meteorol Clim.

[17] Winker DM (2009). Overview of the CALIPSO mission and CALIOP data processing algorithms. J Atmos Ocean Tech.

[18] Varnai, T. & Marshak, A. MODIS observations of enhanced clear sky reflectance near clouds. *Geophys Res Lett* **36** (2009).

[19] Yang W (2016). Observation of the spectrally invariant properties of clouds in cloudy-to-clear transition zones during the MAGIC field campaign. Atmos Res.

[20] Varnai T, Marshak A (2012). Analysis of co-located MODIS and CALIPSO observations near clouds. Atmos Meas Tech.

[21] Varnai T, Marshak A (2011). Global CALIPSO Observations of Aerosol Changes Near Clouds. IEEE Geosci Remote S.

[22] Li R (2014). The optical properties and longwave radiative forcing in the lateral boundary of cirrus cloud. Geophys Res Lett.

[23] Bar-Or RZ, Koren I, Altaratz O, Fredj E (2012). Radiative properties of humidified aerosols in cloudy environment. Atmos Res.

[24] Hunt WH (2009). CALIPSO Lidar Description and Performance Assessment. J Atmos Ocean Tech.

[25] Liu ZY (2009). The CALIPSO L Cloud and Aerosol Discrimination: Version 2 Algorithm and Initial Assessment of Performance. J Atmos Ocean Tech.

[26] Kim SW (2008). Validation of aerosol and cloud layer structures from the space-borne lidar CALIOP using a ground-based lidar in Seoul, Korea. Atmos Chem Phys.

[27] Sassen, K., Wang, Z. & Liu, D. Global distribution of cirrus clouds from CloudSat/Cloud-Aerosol Lidar and Infrared Pathfinder Satellite Observations (CALIPSO) measurements. *J. Geophys. Res*. **113** (2008).

[28] Yang YJ, Lu DR, Fu YF, Chen FJ, Wang Y (2015). Spectral Characteristics of Tropical Anvils Obtained by Combining TRMM Precipitation Radar with Visible and Infrared Scanner Data. Pure Appl Geophys.

[29] Fu YF (2013). Ozone vertical variations during a typhoon derived from the OMI observations and reanalysis data. Chinese Sci Bull.

[30] Xian T, Fu YF (2015). Characteristics of tropopause-penetrating convection determined by TRMM and COSMIC GPS radio occultation measurements. J. Geophys. Res..

[31] Ricchiazzi P, Yang SR, Gautier C, Sowle D (1998). SBDART: A research and teaching software tool for plane-parallell radiative transfer in the Earth’s atmosphere. B Am Meteorol Soc.

[32] Heymsfield AJ, Winker D, van Zadelhoff G (2005). Extinction-ice water content-effective radius algorithms for CALIPSO. Geophys Res Lett.

[33] Wielicki BA (1996). Clouds and the earth’s radiant energy system (CERES): An earth observing system experiment. B Am Meteorol Soc.

[34] Chen T, Rossow WB, Zhang YC (2000). Radiative effects of cloud-type variations. J Climate.

[35] Kienast-Sjogren E (2016). Climatological and radiative properties of midlatitude cirrus clouds derived by automatic evaluation of lidar measurements. Atmos Chem Phys.

[36] Stocker, T. F. *et al*. Climate change 2013: The physical science basis (2014).

